# Enduring Neuroprotective Effect of Subacute Neural Stem Cell Transplantation After Penetrating TBI

**DOI:** 10.3389/fneur.2018.01097

**Published:** 2019-01-17

**Authors:** Anelia A. Y. Kassi, Anil K. Mahavadi, Angelica Clavijo, Daniela Caliz, Stephanie W. Lee, Aminul I. Ahmed, Shoji Yokobori, Zhen Hu, Markus S. Spurlock, Joseph M Wasserman, Karla N. Rivera, Samuel Nodal, Henry R. Powell, Long Di, Rolando Torres, Lai Yee Leung, Andres Mariano Rubiano, Ross M. Bullock, Shyam Gajavelli

**Affiliations:** ^1^Department of Neurological Surgery, The Miami Project to Cure Paralysis, University of Miami Miller School of Medicine, Miami, FL, United States; ^2^Neurosurgery Service, INUB-MEDITECH Research Group, El Bosque University, Bogotá, CO, United States; ^3^Wessex Neurological Centre, University Hospitals Southampton, Southampton, United Kingdom; ^4^Department of Emergency and Critical Care Medicine, Nippon Medical School, Tokyo, Japan; ^5^Department of Neurosurgery, Sun Yat-Sen Memorial Hospital, Sun Yat-Sen University, Guangzhou, China; ^6^Branch of Brain Trauma Neuroprotection and Neurorestoration, Center for Military Psychiatry and Neuroscience, Walter Reed Army Institute of Research, Silver Spring, MD, United States; ^7^Department of Surgery, Uniformed Services University of the Health Sciences, Bethesda, MD, United States

**Keywords:** traumatic brain injury, inflammasome, pyroptosis, neural stem cell, cell transplantation

## Abstract

Traumatic brain injury (TBI) is the largest cause of death and disability of persons under 45 years old, worldwide. Independent of the distribution, outcomes such as disability are associated with huge societal costs. The heterogeneity of TBI and its complicated biological response have helped clarify the limitations of current pharmacological approaches to TBI management. Five decades of effort have made some strides in reducing TBI mortality but little progress has been made to mitigate TBI-induced disability. Lessons learned from the failure of numerous randomized clinical trials and the inability to scale up results from single center clinical trials with neuroprotective agents led to the formation of organizations such as the Neurological Emergencies Treatment Trials (NETT) Network, and international collaborative comparative effectiveness research (CER) to re-orient TBI clinical research. With initiatives such as TRACK-TBI, generating rich and comprehensive human datasets with demographic, clinical, genomic, proteomic, imaging, and detailed outcome data across multiple time points has become the focus of the field in the United States (US). In addition, government institutions such as the US Department of Defense are investing in groups such as Operation Brain Trauma Therapy (OBTT), a multicenter, pre-clinical drug-screening consortium to address the barriers in translation. The consensus from such efforts including “The Lancet Neurology Commission” and current literature is that unmitigated cell death processes, incomplete debris clearance, aberrant neurotoxic immune, and glia cell response induce progressive tissue loss and spatiotemporal magnification of primary TBI. Our analysis suggests that the focus of neuroprotection research needs to shift from protecting dying and injured neurons at acute time points to modulating the aberrant glial response in sub-acute and chronic time points. One unexpected agent with neuroprotective properties that shows promise is transplantation of neural stem cells. In this review we present (i) a short survey of TBI epidemiology and summary of current care, (ii) findings of past neuroprotective clinical trials and possible reasons for failure based upon insights from human and preclinical TBI pathophysiology studies, including our group's inflammation-centered approach, (iii) the unmet need of TBI and unproven treatments and lastly, (iv) present evidence to support the rationale for sub-acute neural stem cell therapy to mediate enduring neuroprotection.

## Introduction

TBI is a critical public health problem and one of the leading causes of death and disability around the globe (1–5). The World Health Organization (WHO) and the World Bank estimate that 69 million (95% CI 64–74 million) individuals suffer from TBI every year, with Southeast Asian and Western Pacific regions experiencing the greatest overall burden (6). A recent estimate of the Global Incidence of TBI puts it at ~939 cases per 100,000 people each year with 79% being mild TBI. The calculated incidence of TBI in the Americas (including United States (US) /Canada) is 1,299 cases per 100,000 people each year. The calculated incidence for Latin America is about 909 per 100,000 people each year (7). Worldwide about 90% of all TBI-related deaths occur in developing countries (8). In 2016, road traffic injuries were among the three leading causes of death from injuries independent of gender. The economic status (a surrogate for investment in health care, trauma centers, and road safety) of the country rather than its global location appear to influence trauma outcomes. For example, in the poorest country in the Western Hemisphere, road traffic accidents accounted for >40% of TBI incidence (9). Similarly, African and Eastern Mediterranean regions are above the global average while the rest are on par or below (6, 10). Within the US, road traffic accidents have been on the decline and apart from age related vulnerability to falls, firearm injury has become an increasingly serious problem (11, 12). Overall, TBI affects 1.7 million people in the US with ~50,000 fatalities annually. Timely and aggressive management of acute trauma patients has lowered the fatality rate but does not eliminate the socioeconomic consequences of TBI (13–17). The annual cost of TBI in the US is estimated to be between $168 billion in medical spending and $223 billion in work losses (18). Globally it is estimated at $400 billion (19). Despite the outpouring of resources for TBI management and research, 5.3 million TBI patients in the US continue to live with disabilities, a consequence that is independent of injury severity (20). Improved clinical care has led to increased post injury survival, while return-to-work has remained static for the past five decades (21–24). The current clinical management of severe TBI exploits the limits of physiological interventions and addresses issues mainly at the systemic level and sometimes at the cellular and biochemical levels but rarely at the subcellular organelle dysfunction level. As an example, TBI induced mitochondrial dysfunction has remained intractable (25). Consequently, TBI survivors experience the full wrath of secondary mechanisms (26–30). This “secondary mechanism fueled” histopathology seen in human TBI is recapitulated with preclinical TBI models (31–36) and offers an opportunity to test interventions.

## Current TBI Treatment

The Brain Trauma Foundation (BTF), a non-profit group of TBI expert clinicians, has dictated the management of severe TBI since its establishment in 1996 (37, 38). Since that time, there has not been much change in the treatment of TBI despite a better understanding of the destructive events inherent to the disease process. Though adherence to these guidelines decreased overall healthcare costs and improved patient survival (39, 40), the latest fourth edition offers no class I and few class II recommendations in regards to severe TBI management. The major focus of current neurointensive care is (i) metabolic stabilization of the patient, (ii) prevention of further deterioration, and (iii) facilitation of “spontaneous” brain recovery. Along with prompt neurosurgical interventions when warranted, optimizing hemostasis, oxygenation, ventilation, temperature, blood pressure, blood glucose, and acute seizure prophylaxis increased positive outcomes after severe TBI (38, 41, 42). Contrary to previous guidelines, a Glasgow Coma Scale (GCS) of lower than 5 is no longer a contraindication to surgery because of advances in modern surgery and the neurointensive care unit which have improved survival of these patients (43, 44). Early management and proper monitoring of parameters such as intracranial pressure and sodium levels have limited certain types of secondary brain injury (42, 45). Compliance with BTF guidelines is proportional to the strength of evidence (46). For implementation of an efficient trauma system in under-privileged areas, the organization of low cost resources such as trauma registries are required (47–50). For example, Latin American neurosurgeons have advocated for improving clinical research methodologies and topics in the region (51), to better understand implications and relationships between intervention and outcomes. Aggressive surgical therapy seems to be an option for improving survival even in penetrating TBI (PTBI) (48) in developed countries. Intensive critical care management and less aggressive surgical therapy based on the military experience acquired during the 1970's war in Lebanon also produces favorable outcomes especially in pediatric and adult severe TBI (52, 53). Severe TBI patients are treated with a combined medical-surgical approach, managed initially in the intensive care unit (ICU) with neuromonitoring (54, 55), in conjunction with BTF “living” guidelines (updated to incorporate the findings of randomized clinical trials (RCT), the gold standard for proving the efficacy of new treatments) (37, 38, 56). The next section revisits a few trials and identifies TBI pathophysiological processes that may have led to their failure.

## Can Insights Into TBI Pathomechanism Explain Failure Of Past Neuroprotection Trials?

**Figure 1:**
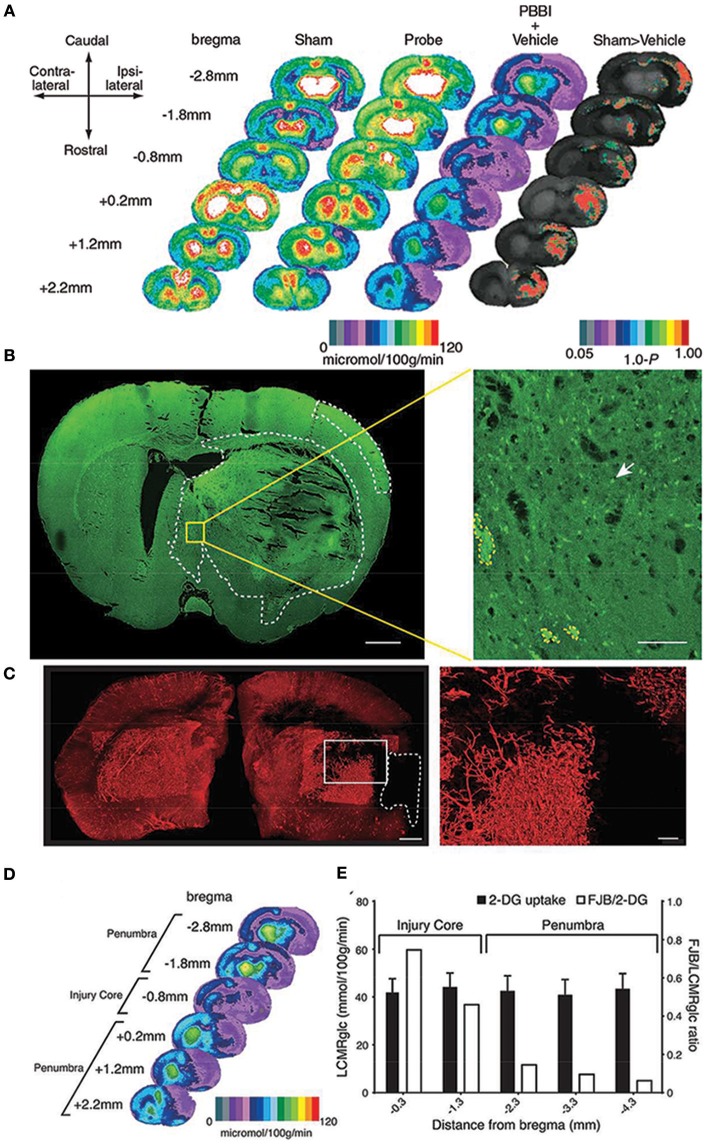
Local cerebral glucose metabolism after penetrating ballistic-like brain injury (PBBI) **(A)** is shown as color-coded maps of average local cerebral metabolic rate for glucose (LCMRglc) at 2.5 h after injury. Each coronal section is a representation of multiple animals within a group at that particular level. Rat brain atlas levels are given on the left column as millimeters from bregma. Compared with controls (columns 1 and 2) in PBBI (column 3), LCMRglc decreased radially from injury core into perilesional areas and globally across the entire brain. P-maps of average local cerebral glucose utilization were produced by comparing the values of pixels corresponding to the same anatomic position across groups. **(B)** Confocal image of a Fluorojade B (FJB)-stained coronal section at 0.8 mm distance from bregma shows regions with FJB+ cells (circumscribed by white-dotted line). Greater neurodegeneration was observed in the injury core and peri-injury zone in the ipsilateral than those in the contralateral cerebral cortex. **(C)** Composite light sheet microscopy image shows ipsi and contralateral hemispheres perfused with fluorescent tomato-lectin at 2.5 h post PBBI. Region with injury induced hypoperfusion is circumscribed by white-dashed line. Surface reconstruction renders the labeled vasculature in 3D. **(D)** Hypoperfused region overlaps with the 2-deoxy glucose (2-DG) uptake impairment heat map. **(E)** The incidence of neurodegeneration was proportional to 2-DG uptake impairment at the injury core but not in regions caudal to the injury core. Fluorojade B (FJB)/LCMRglc ratio decreased from injury core toward more caudal regions, decreasing maximally at−2.3 mm from bregma and plateaued (penumbra). Further details are present in the original article (78).

Primary injury, which occurs at the time of impact, includes tissue laceration, cerebral contusion, axonal damage and hemorrhage. Following hospital care, TBI patients can also remain disabled, rendering them worse off which led to the conclusion that secondary injury. It was deduced that secondary insults also significantly influenced outcomes (20, 56, 57). Investigations into the secondary injury process revealed several concurrent processes with distinct spatiotemporal peaks occurring within seconds after injury and lasting for years (58, 59). The result is a complex cascade of molecular and cellular damage, which magnifies the primary injury causing delayed and remote secondary injury (60–64). Initial descriptions of secondary mechanisms included clinical parameters necessary for decision-making, which led to the invention and adoption of the Glasgow coma scale (GCS) (57, 65). The list now includes parameters known to influence TBI outcome such as cerebral blood flow (66), hypoxia-ischemia (67), mitochondrial dysfunction (25), cerebral metabolism (68), cell death (69), glutamate excitotoxicity (70, 71), calcium dysregulation, edema (72) culminating in inflammation, the most enduring of the secondary damage mechanisms (73–75). Inflammation has also been linked to depression like symptoms causing depression like symptoms *via* failure of neurogenesis (76, 77) in multiple CNS conditions including TBI. All these processes have been recapitulated in animals model (Figures 1) (78). In the early post-traumatic period (seconds to days), injured neurons in contusions appear swollen, but over time (days or weeks), they become shrunken and eosinophilic, with pyknosis of the nuclei (79). Neuronal and glial “apoptosis” was observed after TBI in human tissue prior to description of the process (69) and later confirmed (80).

Over the three decades, the improved survival of TBI patients upon management with Glasgow coma score (21, 65) and the adoption of cerebral cardiopulmonary resuscitation (CCPR) protocols based upon quantitation of physiological measures (81) led to RCTs that attempted to block/reverse the TBI pathological processes. Such RCTs mostly failed to yield any class I evidence necessary to improve TBI outcomes. These trials included surgical interventions, which unlike decompressive craniectomy (DC) in stroke (82), did not find benefit and had to be stopped due to adverse effects and low recruitment. For e.g., both Decompressive Craniectomy in Patients with Severe Traumatic Brain Injury (DECRA) and Randomised Evaluation of Surgery with Craniectomy for Uncontrollable Elevation of Intracranial Pressure (RESCUEicp) showed poor outcome (55). DECRA was criticized for excluding second tier treatments often used in “real life,” not representing the “real world population,” and because the duration of high ICP was too short (83, 84). Further negating the DECRA findings, a retrospective analysis revealed benefit of DC and/or barbiturate combination for refractory intracranial pressure management after severe TBI (85). More recently another DC trial (with 80% of patients similar to DECRA and 38% to RESCUE-ICP) showed that the addition of a barbiturate step following DC was more effective than DC alone, barbiturate alone or barbiturate before DC (86). RESCUEicp reported that at 6 months post-decompressive craniectomy, mortality was lowered but at the cost of higher rates of vegetative state, and severe disability. The trial evaluating Early Surgery vs. Initial Conservative Treatment in Patients with Traumatic Intracerebral Hemorrhage was halted after enrolling <20% of the planned number (87, 88). The limited success of surgical intervention is unsurprising as numerous secondary processes (discussed below) are initiated after primary insult and cannot be surgically targeted. More perplexing is the failure of neuroprotective pharmacological RCTs (11, 89–92) including the ProTECT trial (93) which were based on robust preclinical data. Therefore, in the next section we explore the possible reasons that single TBI pathological mechanism targeting RCTs failed.

### Mitochondrial Dysfunction/Calcium Dysregulation

TBI induced mitochondrial dysfunction is the rate-limiting step in metabolic restoration of a patient with clinical management (25, 94, 95). Persistently elevated intracellular calcium levels play a central role in activating cellular death mechanisms. Dysfunction of mitochondria (96), production of pro-inflammatory cytokines, as well as axonopathy (97) are all related to calcium dysregulation (98). Upon binding of calcium, the calmodulin-calcineurin complex upregulates the expression of IL-2 by activating the transcription factor NFAT. IL-2 stimulates the proliferation of T lymphocytes, which then recruit more immune cells and amplify the process (99). This pathway is exploited in the treatment of cancer, transplant rejection, and autoimmune diseases. Insights into what constitutes mitochondrial dysfunction came from studies in cardiomyocytes. In these cells low ATP, high calcium caused mitochondrial dysfunction due to the opening of high conductance pores in the inner mitochondrial membrane, uncoupling mitochondrial oxidative phosphorylation and promoting ATP hydrolysis. Cyclosporine A (CsA) was found to prevent such pore opening in isolated mitochondria (100) and cells (101). However, prevention of neurological deterioration was incorrectly attributed to CsA (102), albeit unknown at that time (103). CsA was found to be beneficial in transient forebrain ischemia rodent models upon intracerebral injection provided it could cross the blood brain barrier (BBB) (104). It was therefore given before and after the injury to enable entry to the brain during the opening of BBB and stabilized isolated mitochondria in rodent TBI brains (105). However, despite safety in humans (106, 107), the drug failed to meet the OBTT criteria for advancing to translation (108). This could be in part due to inadequate dosing or possible adverse effects associated with the vehicle Cremaphor. The drug also has a short therapeutic window and needs continuous infusion over the first 3 days post injury to stabilize the mitochondria (109–112). Recent work with a new carrier in gyrencephalic animals is reportedly neuroprotective (113). A related drug, minocycline, although not tested in OBTT and initially used for a different purpose i.e., reducing neuroinflammation via ablation of activated microglia, was tested in human TBI and found to have no benefits (114) though the marker of inflammation was reduced. Minocycline, a well-known antibiotic in the tetracycline family with anti-inflammatory qualities that include inhibition of NFkB prevents the transcription of pro-inflammatory cytokines and activation of M1 microglia (99, 115). Consistent with equivocal data in rodent TBI (116), a clinical trial using minocycline in chronic TBI patients (6 to 142 months post TBI) showed reduced microglial activation but increased neurodegeneration over time (114). Microglial activation, measured with ^11^C-PBR28 PET was reduced after 12 weeks of treatment with minocycline but plasma axonal neurofilament light levels, a marker of neurodegeneration, increased and white matter atrophy was more prominent on MRI (114). This suggests that mitochondrial function in even activated microglia are necessary to prevent neurodegeneration, and that there is a need to separate the anti-inflammatory regenerative properties e.g., phagocytosis and proinflammatory degenerative properties e.g., pyroptosis that reside within the same activated microglia, rather than merely ablating all microglia. It is possible that attempts to reduce proinflammatory activated microglia via cell ablation does not mitigate their pyroptosis, an inflammatory process that causes damage to intact tissue. Instead there may be a need to ramp up the anti-inflammatory microglia activity (117). Collectively, preclinical data and the minocycline TBI trial suggest that non-specifically targeting activated glia through these corresponding classes of drugs [Consistent with such data mitochondrial uncoupling agents are also capable of tissue sparing (118)] is not sufficient for neuroprotection in neither an acute nor chronic time point when used with certain dosing regimen.

Calcium is also required for the activation of calpain proteases that cause the breakdown of the cytoskeleton and the cessation of axonal transport (97). Neurofilaments and microtubules accumulate and swelling ensues, forming axonal bulbs that eventually separate the axon. Years after injury, extensive axonopathy is associated with atrophy of the brain, expansion of the ventricles, and premature dementia (119). Of the several clinical trials that evaluated the effect of the calcium channel blocker nimodipine in acute severe TBI, only one improved neurological outcome at 6 months (120). However, subgroup analyses from failed studies revealed that the patients with evidence of subarachnoid hemorrhages on CT benefited from the drug (121). Nimodipine is now widely used in patients with SAH from traumatic and non-traumatic etiology as vasospasm prophylaxis.

### Glutamate Excitotoxicity

Another contributory mechanism to TBI is a surge of excitotoxic amino acids, primarily glutamate, that occurs which causes irreparable disturbances in ion fluxes (71). Binding of glutamate to the N-methyl-D-aspartate (NMDA) receptor results in depolarization of neurons, followed by a massive influx of calcium, and efflux of (71, 122). Loss of GABAergic inhibition (possibly due to higher numbers of GluR3 subunit containing but GluR2 subunit depleted AMPA receptors which allow influx of calcium mediating greater vulnerability to excitotoxicity), and downregulation of astrocytic glutamate transporters which allow excitatory neurotransmitter accumulation in the synapse culminating in neuronal and glial (oligodendrocyte) cell death (123, 124). The integrity of GABAergic inhibition needed to contend with excitotoxicity may be compromised by reduced glutathione activity. Glutathione activity was reduced in a rodent model of TBI (125), and increased super oxide production decreased parvalbumin expression in parvalbumin (PV+) GABAergic cells (126). PV+ cells have dendritic arborizations receiving fast converging excitatory inputs (127–130) as reviewed earlier (131) and are endowed with a fast spiking phenotype (132). Higher cognitive functions such as perception (133), and the deterrence of epileptiform activity (134, 135) is contingent upon reliable PV+ cell mediated inhibition. As such, pharmacological disruption of glutamatergic signaling onto fast spiking parvalbumin GABAergic cells disinhibits the circuitry mediating gamma oscillations which facilitate information storage and transfer (136). Promising preclinical results with magnesium blockade of the neuronal NMDA receptor (70), did not translate into neuroprotection in clinical trials, in part due to narrow therapeutic windows, adverse side effects, and interference with normal electrical activity of the brain (11, 19, 90, 137). Glutamate NMDA receptor antagonists (competitive receptor antagonists, ion channel blockers, and glycine antagonists)—such as selfotel, aptiganel, eliprodil, licostinel, and gavestinel—failed to show efficacy in clinical trials of stroke or traumatic brain injury. Deficient properties of the molecules used in human trials or inappropriate design of clinical studies may have contributed to failure. It is possible that excitoxicity kills inhibitory GABAergic cells (such as hippocampal parvalbumin neurons) due to their higher amounts of GluR3 containing and GluR2 lacking AMPA receptor subunits than glutamatergic neurons, as mentioned above. The clinical NMDA antagonists dose then targets only the glutamatergic neurons, oligodendrocytes, and astrocytes impairing brain metabolic capacity. An alternative hypothesis suggests that glutamate may be involved in the acute neuro-destructive phase immediately after traumatic or ischaemic injury (excitotoxicity), but later, is required for normal physiological functions except during spreading depolarizations (138). Thus blockade of synaptic transmission mediated by NMDA receptors may hinder neuronal survival (139). Ikonomidou speculated that these drugs could be useful if used just prior to a TBI event, akin to an unexpected decreased in TBI related mortality in alcohol and methamphetamine abusers (140, 141), despite neurotoxicity of the abused substance (142). It remains to be seen what role such drugs can play in TBI management.

### Hyperglycolysis

Human brain normally uses glucose as the sole substrate and due to lack of fuel stores; the brain requires a continuous supply. Seymour Kety and Carl Schmidt introduced the first quantitative measurements of human, whole-brain blood flow and metabolism (143). Kety, Sokoloff and their colleagues noted that, while the human brain is only 2% of the body weight, it accounts for 20% of the body's energy consumption. Their technique laid the foundation for studies of brain metabolism in terms of rates of glucose and oxygen consumption (144). When the technique was applied to TBI, both cerebral metabolic rate of oxygen (CMRO_2_) and cerebral metabolic rate of glucose changes (CMRO_2_) were evident (66, 145). During the first 6 days after moderate or severe TBI, CMRO_2_ and arterial lactate levels are the strongest predictors of neurologic outcome (146). Relative to the impaired glucose uptake in the TBI brain, some regions exhibited a high level of glycolysis. This process called “hyperglycolysis” is defined as an increase in glucose utilization two standard deviations above the normal. Hyperglycolysis is thought to be mediated by the release of catecholamines in response to injury to meet increased energy demand upon cells to drive pumping mechanisms in order to restore membrane ionic balance (147). In a study of 28 patients, Hovda et al. reported that hyperglycolysis was observed in 56% of patients on fluorodeoxyglucose-positron emission tomography (FDG-PET within the first week of injury and persisted for several weeks (148, 149). In the context of TBI, it is not clear which cells are undergoing hyperglycolysis. Lactate accumulation in the injured brain can stem from neuronal mitochondrial dysfunction (150) and/or due to massive influx of lactate from peripheral tissues (151). In turn, elevated lactate levels contribute to pan necrosis (plasma membrane damage, cerebral edema, BBB permeability and overall cell breakdown) (152). In preclinical (153) and clinical studies (149) of TBI, hyperglycolysis and related metabolic crisis (non-ischemic high lactate) increased the incidence of spreading depolarization (138), seizures (154) and were associated with poor outcomes (148). Misled by an “insufficient fuel” concept, glucose supplementation was explored in preclinical studies but surprisingly turned out to be harmful (155–157). However, as in the case of mild TBI, mere fasting was found to be neuroprotective (158). Accordingly, there is no consensus on glycemic control after TBI. Novel metabolic imaging techniques (159) or combination of metabolic studies and neuromonitoring with imaging will be key to gaining insights into the TBI metabolic crisis (94, 138, 150, 154). In three clinical trials, intensive insulin therapy—when compared to conventional insulin therapy—consistently increased the risk of hypoglycemia in moderate to severe TBI patients and failed to decrease mortality at 6 months (160–162). Among these studies, only Yang et al. reported better neurological outcome, measured with Glasgow Outcome Scale (GOS), at 6 months. Supply-demand mismatch, generated from increased metabolism in the setting of decreased cerebral blood flow (CBF), provokes an energy crisis that promotes further damage (94).

This led to the search for alternative substrate to improve cellular metabolism. Ketogenic diet (KD) via induction of ketosis is known to increase cerebral metabolism of ketones. Age-dependent neuroprotection after TBI in part could be due to younger animals achieving significant β-hydroxybutyrate levels earlier than adults do. In both juvenile rats subjected to weight drop model and adolescent rats to cortical contusion injury (CCI), KD resulted in decreased brain edema, cytochrome c release, apoptotic and oxidative stress marker expression, mitochondrial calcium loading, improved cellular energetics, increased expression of brain-derived neurotrophic factor, smaller contusion volumes and better motor, and cognitive performances. Ketosis mediated by fasting or calorie restriction was also neuroprotective in adult rats with TBI. One of the prominent mechanisms of KD includes inhibition of glycolysis (and subsequently dependent proinflammatory cytokine synthesis), thus lowering inflammation and upregulating bioenergetics via mitochondrial biogenesis (163). At the organelle level a ketogenic diet was found to reduce onset of seizures by preventing the opening of mitochondrial membrane permeability transition pores (164) effectively acting as a neuroprotective uncoupling agent (165).

In contrast to the beneficial effects of ketone metabolism, poor nutritional support can exacerbate TBI (166). Currently, clinical studies are underway to determine the optimal method to induce cerebral ketone metabolism in the post-injury brain, and to validate the neuroprotective benefits of ketogenic therapy in humans (167). Improvements in the understanding of human brain metabolism (168) led to the documentation of metabolism perturbations in injured brains (94, 169–171) and the ability to test if supplementation can bypass these impairments (172).

### Hypoxia

In order to maintain its high metabolic activity, the brain receives a substantial proportion of the cardiac output and is therefore highly susceptible to hypoxia (173, 174). Under normal circumstances, a decrease in arterial partial oxygen tension (pBTiO_2_) is balanced by increases in cerebral blood flow (CBF) to prevent cerebral ischemia, sometimes at the expense of rising ICP (175). In TBI, loss of autoregulation, as evidenced by concurrent reduction in CBF and pBTiO_2_, is one of the mechanisms that exacerbate injury. Hypoxia accelerates uncoupling of the electron transport chain and mitochondrial permeabilization, which induces the release of pro-apoptotic signals, such as reactive oxygen species (ROS) and cytochrome c, inside the cytosol (96). Leakage of cytochrome c in conjunction with elevated cytoplasmic calcium activates the caspase cascade, leading to cell apoptosis. HIF-1α is a constitutively expressed protein whose activity depends on oxygen availability (176). In normoxia, HIF-1α is hydroxylated by prolyl hydroxylase (Lee et al.) then tagged with ubiquitin for degradation in proteasomes (177). In contrast, hypoxia decreases the activity of PHD and HIF-1α is translocated to the nucleus where it binds HIF-1β and pyruvate kinase M2. This complex induces the transcription of molecules that stimulate the expression of genes involved in glycolysis, angiogenesis, neurogenesis and the synthesis of proinflammatory cytokines (177–179).

Methods to alleviate TBI-induced brain tissue hypoxia by using blood substitutes, increasing hemoglobin, oxygen saturation, or oxygen tension are currently part of the TBI critical care armament. Attempts to improve cerebral oxygenation with blood substitutes such as perfluorocarbons (PFCs) alleviated hypoxia in animal models of PBBI (78, 180, 181). However, clinical translation of this artificial oxygen carrier was deemed unsafe due to the development of thrombocytopenia, an abnormality that could be detrimental in TBI patients, which led to the cessation of the Safety and Tolerability of Oxycyte in Patients With Traumatic Brain Injury (STOP-TBI) trial (182). Insights into PFC cellular distribution facilitated its repurposing to instead identify injury penumbra: perfluorocarbon enhanced Glasgow Oxygen Level Dependent (GOLD) magnetic resonance metabolic imaging (183).

Because of the prevalence of anemia in TBI patients and associated worse outcomes, administration of erythropoietin (EPO) was evaluated after head injury. The discovery of EPO, a hormone principally produced by the peritubular interstitial cells of the kidney, revolutionized the treatment of anemia in chronic kidney disease patients. Identification of EPO of neuronal and astrocytic origins and the hormone's non-hematopoietic functions (184) have been investigated. Binding to the EPO receptor prevents apoptosis of mature neurons and enhances the proliferation of neural progenitor cells (185). Its anti-apoptotic properties are mediated by the inhibition of pro-apoptotic molecules -including apoptosis regulators bcl-2-like protein 4 (BAX) and cytochrome c, and the activation of the NFKB pathway, which results in the stimulation of the adaptive immune system cells. EPO also promotes proliferation of the endothelium and the production of nitric oxide (NO) (185). In the multicenter international EPO-TBI trial, EPO did not display neuroprotective effects in patients with moderate to severe TBI (186, 187). Perhaps EPO-induced inflammatory NO may have abrogated any beneficial effect as it increased lesion volume after PBBI (188).

Although, it could seem counterintuitive to use hyperoxia in TBI (189) (i.e., to avoid reperfusion injury), researchers found that markedly increasing oxygen (O_2_) delivery to the traumatized brain, with hyperbaric oxygen therapy (HBOT) or normobaric hyperoxia (NBH), could reverse the lack of O_2_ for e.g., vascular stenosis (Figure 1) that precipitates cellular energy failure and subsequent neuronal death. A recently published review identified eight phase I and phase II clinical trials evaluating the role of acute and subacute HBOT and/or NBH in severe TBI patients. Overall, HBOT alone or in combination with NBH improved physiologic markers of metabolic function (microdialysate LPR, glycerol, ICP) and decreased long-term morbidity and mortality to a greater extent than NBH alone or standard of care (67, 190–192). The “Hyperbaric Oxygen Brain Injury Treatment” (HOBIT) trial: (193) is a proposed adaptive clinical trial designed to answer questions about dosage and safety parameters of HBO_2_ and to provide important data to plan a definitive phase III efficacy trial.

### Edema

After TBI, edema develops because of cellular dysfunction (cytotoxic edema) and blood brain barrier (BBB) disruption (vasogenic edema). Increased permeability of the cell membrane to Na+ and K+ followed by failure of the Na+/K+ ATPase pump traps osmotically active molecules inside the cell. Mechanical destruction of endothelial cells causes the capillaries to leak a protein-rich exudate into the brain parenchyma. CBF reduction, glutamate excitotoxicity, osmolar gradients additionally participate in extending the edematous state and can contribute to elevations in ICP (194). Different osmotic therapies (mannitol, hypertonic saline, hypertonic lactate, barbiturate) have been examined but none have improved long-term neurological outcome or survival (38, 186). The “BRAIN” trial tried to exploit the role of the kallikrein-kinin system in TBI but it was terminated because Anatibant, an antagonist of the bradykinin B2 receptor, caused more deaths than control 15 days post-injury (195, 196). The kinin family also is known to have a neuroprotective role via the attenuation of microglial proinflammatory secretion through actions of prostaglandin E and microsomal prostaglandin E synthase (197, 198). Bradykinin receptor B1 but not B2 deficiency protects from focal closed head injury in mice by reducing axonal damage and astroglia activation (198, 199). Anatibant may have selectively inhibited the neuroprotective effects while allowing proinflammatory signaling to persist leading to poor outcomes. Nevertheless, the historical failure of acute neuroprotective interventions (11, 114, 137, 200–202) has exposed the limitations of preclinical TBI models in guiding clinical trials in TBI. Similarly, limitations inherent in pre-clinical testing such as insufficient rigor in pre-clinical studies may also have contributed to RCT failure (203). To offset this two groups have come up a similar suggestion regarding data reporting in preclinical studies that would help compare preclinical studies. Use of “delta Score” i.e., summing the change in outcome that may occur in patients in the placebo vs. drug-treated groups over time or effect size used to run meta-analyses are helpful (204, 205). In aggregate, the RCTs failed as they allow for persistence of dual edged inflammation. To provide insights into how unmitigated inflammation underlies progressive tissue loss, our laboratory research uses a rodent model of penetrating TBI (PTBI). PTBI and TBI secondary damage mechanism are similar and may differ only in magnitude. Acute and delayed consequences of human PTBI (64, 206–208) are replicated in Penetrating ballistic-like brain injury (PBBI), a survivable rat PTBI model (34, 35, 78, 209, 210). We detail the results from using this model below.

## Why Is The Penumbra Vulnerable?

**Figure 2:**
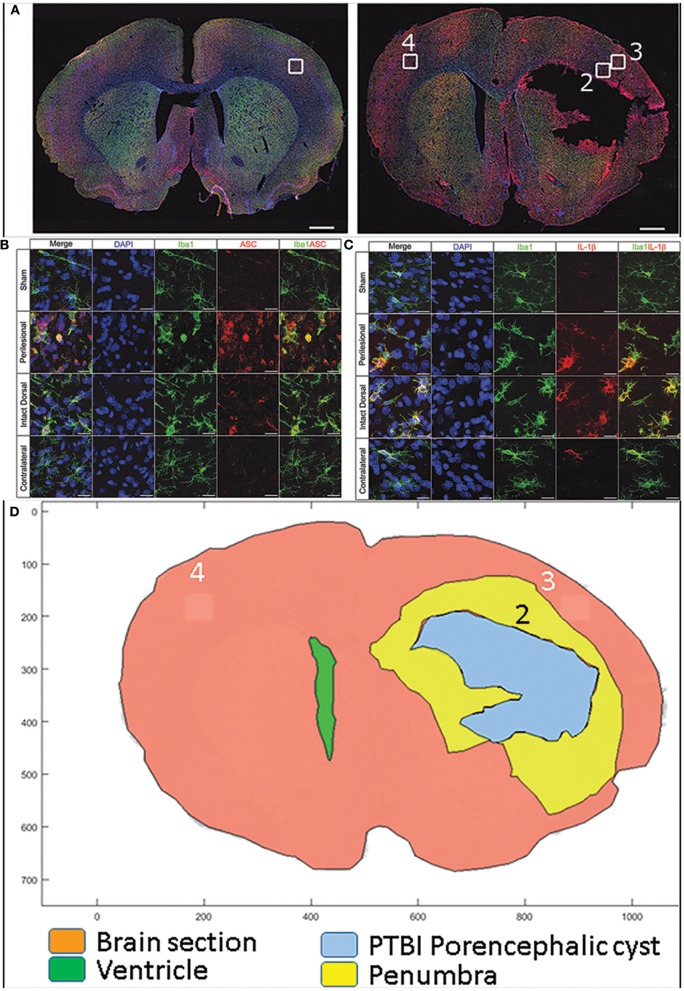
Confocal images of free-floating rat brain sections stained with 2-(4-amidnophenyl)-1H-indole-6-carboxmidine (DAPI; blue), ionized calcium-binding adapter molecule 1 (Iba-1; green), and apoptosis speck-like protein containing caspase-activation and recruitment domain (ASC) or interleukin (IL)-1b (red). Top panels **(A)** show whole–brain sections from a representative sham (left) and 10% penetrating ballistic-like brain injury (PBBI) animal 48 h after injury (right). Sections show Iba-1+ microglia widely dispersed throughout the brain. White boxes (1–4) in the whole–brain images are shown at 100x magnification in panels below. ASC immunoreactive cells are absent in sham cortex (box 1), numerous ASC positive cells are present in PBBI perilesional area (box 2), but to a lesser extent in PBBI intact ipsilateral dorsal cortex (box 3), and absent in contralateral cortex (box 4) **(B)** Iba-1 and ASC double positive cells are present in the ipsilateral hemisphere. In **(C)** Iba-1 and IL-1b co-labeled cells are predominantly present in the ipsilateral cortex. Double positive cells are morphologically large with round/hypertrophied cell bodies and short processes. Additional details are presented in the original publication (210). By subtracting the traces of the brain sections at 48 h post PBBI from those at 10 weeks post PBBI, the PBBI penumbra (box 2 within yellow highlight) is identified **(D)**. The penumbra (box2) was occupied by highly activated microglia at 48 h post injury is lost by 10 weeks post PBBI. In contrast, in box 3 microglial were activated to a lesser extent and at 10 weeks post injury such tissue survives.

Our recent study with rat PTBI showed that the ipsilateral cortical region at 48 h post injury is replete with activated microglia (boxed regions in Figures 2A–C) (210). Only the “penumbra” (yellow highlight in Figure 2D) disappears by 10 weeks post injury (35) while regions more dorsal persist despite the presence of proinflammatory activated glia (210). It is possible that a gradient of DAMPS or NAMPs exists with the high concentration at the injury core gradually diminishing radially in a tissue architecture dependent manner. Consistent with this concept, activated microglia in penumbra surrounding the core region may undergo pyroptosis unlike microglia in distal regions. Consequently by 10 weeks post injury the entire penumbra with “critical” levels of DAMPs/NAMPs” disappears while the regions with sub-critical levels survive. This data is consistent with human TBI study where molecular characterization revealed greater numbers of activated microglia in pericontusion (penumbra) than contusion (211). Hence, if reversal of penumbra vulnerability changes TBI outcomes, rescuing the tissue may become a priority.

## Vulnerable TBI Events That Can And Need To Be Targeted By Clinical Therapy To Spare Penumbra

### Inflammation

Within minutes of injury, damaged cells release damage-associated molecular patterns (DAMPs)—high extracellular potassium, adenosine triphosphate (ATP), mitochondrial DNA, heat-shock proteins (HSPs), high mobility group binding proteins (HMGB1) molecules, and Amyloid beta. These can activate an inflammatory response in nearby cells (212, 213). Consequentially, the assembly of inflammasomes, activation of complement pathways and local immune cells, and production of pro-inflammatory chemicals (chemokines, cytokines, ROS, NO) trigger inflammatory cell death mechanisms (79, 210, 214). The production of interleukin 1 beta (IL-1β) by activated microglia peaks 48 h post-injury and favors polarization of microglia into the pro-inflammatory type.

Broad anti-inflammatory interventions such as hypothermia (215) or neuropeptide blockade (216) appear to be promising based on biomarker profiles. The anti-apoptotic and anti-inflammatory effects of hypothermia have also been investigated in TBI (217). Use of hypothermia for refractory ICP after TBI was beneficial in some centers (217). Particularly in China where three of the four trials had positive effects (acute reduction in ICP and long-term improvement of neurological deficits and mortality at 6 months), however all other trials failed to show similar results (186) e.g., Eurotherm3235 Trial, POLAR RCT failed to reproduce the benefits and stopped due to adverse effects. In the Eurotherm trial, titration with therapeutic hypothermia successfully reduced ICP in participants with TBI + ICP of >20 mmHg, but also led to a higher mortality rate and worse functional outcome (218). *Post-hoc* subgroup analysis of the NABIS-HII trial revealed that hypothermia improved outcomes in patients with evacuated subdural hematomas (219). The failure of the latest hypothermia trial in TBI (220) provides insights into the barriers of translating preclinical findings into human TBI and may unfortunately lead to suboptimal use of this potentially powerful therapeutic in potentially treatable severe trauma patients (221). However, anti-inflammation is not the only consequence of hypothermia, as this approach continues to remain controversial in TBI due to its risk of altering mortality or leading to poor outcomes or new pneumonia (222, 223).

Based on anti-inflammatory action in rheumatoid arthritis, anakinra, FDA approved competitive inhibitor of an interleukin 1 (IL-1) receptor, role of such signaling was evaluated in controlled cortical impact (CCI), a TBI model in rodents, injured IL-1R1 null and wild type mice did not differ in respect to brain lymphocyte numbers (224). In a less sever TBI model, ablation of ILR1 signaling or exogenous IL-1Ra was sufficient to reverse TBI induced deficits (225). Both IL1-alpha (IL-1α), IL-1β signal through the same IL1R. Elevated IL1alpha/IL1 beta are associated with favorable outcomes after TBI (226, 227). Off label, use of anakinra for human TBI is reportedly beneficial (228) in that it shift the microglia to less inflammatory phenotype (229). Based on the preclinical data the detrimental effects of IL1R signaling seem to dominate over the beneficial effects in TBI context hence seeking total IL1R blockade in human TBI needs to be tested next. Another inflammatory cytokine of interest is tumor necrosis factor alpha (TNF-alpha), which upon interacting with TNF receptor 1 but not TNF receptor 2 was found detrimental in neurodegenerative disorders (230). This made an FDA approved TNF antagonist Etanercept, an attractive candidate for decreasing microglia activation after human TBI (231). However, further studies are needed to achieve selective blocking of the TNF receptor 1 rather than broad TNF receptor blockade.

Example of an anti-inflammatory not useful in TBI is statin. Statins downregulate the expression of vascular adhesion molecules and chemoattractant molecules, and were thought to be potential candidates in lowering the infiltration by immune cells into injured brain. However, in a clinical trial, administration of rosuvastatin 11 h after injury did not display any differences in terms of disability (amnesia and disorientation time) with the control group at 3 months but increased IL-6 levels were seen 3 days after injury (232). Consistent with these results, the OBTT study found no beneficial effects of simvastatin administration over 2 weeks post-TBI using the oral route of administration in multiple rodent models (233). Statins are known to inhibit mitochondrial complex III (234) and can produce myopathy as a side effect (235). Thus, statins possibly exacerbate TBI mitochondrial dysfuction (95) which may be the reason that they failed to provide any benefit.

Although on the surface it appears that since inflammation after TBI and SCI are mediated by activated microglia these two pathologies could be identical, there is evidence to suggest that the extent of mitochondrial impairment (a measure of inflammation amplification) is different (236), which becomes apparent with aging. For example, a neuroinflammatory modulator, FTY720, which was unable to improve lesion size or functional outcome in both trauma models at either stage, acute vs. chronic, when given as a single dose (237), improved neurological outcome when dosed over 3 days as was seen with CsA (230), and was more effective in SCI even though the inflammation in SCI is different from that in TBI (238).

While microglia are major effectors of inflammation and mediated neuronal death, neutrophils are the first peripheral immune cells to reach the site of injury (239). Over the following hours to days after trauma, neutrophils infiltrate the CNS and migrate across the BBB in response to chemoattractants secreted by the choroid plexus (99). Recruitment of monocyte-derived macrophages and T lymphocytes then follows. Antibody blockade of cluster of differentiation (Cd) Cd11d/CD18, a type of integrin found on both neutrophils and macrophages, reduced systemic inflammatory response syndrome and improved neurological outcomes in rodent models of TBI (240, 241). In contrast to microglia, circulating immune cells such as neutrophils in TBI only produce short duration inflammation that resolves in part due to gasdermin (242). It remains to be shown if this molecule can be exploited to resolve TBI induced microglial inflammatory response.

The role of the adaptive immune system in TBI is still unclear. While infiltration of T cells in the lesion site promoted inflammation in a rat model of SCI, T cell-deficient mice were found to have poorer outcomes than controls after CNS injury (243, 244). Though T cells could have neuroprotective function in TBI, maladaptive response to self-antigens in conjunction with M1-like microglia action can extend damage and maintain a chronic state of low-level inflammation (117).

Inflammation modulation could reduce the loss of neurons, oligodendrocytes, and neural stem cells. In addition, clearance of debris could help resolve and prevent secondary tissue loss. This approach has been found to mitigate injury-induced cognitive decline at 3 weeks post TBI. Inflammation reduction by suppressing polarization into pro-inflammatory microglia (115, 116), promoting anti-inflammatory microglial activity (245) or enhancing clearance of apoptotic cells (246) may confer greater neuroprotection than focusing solely on inhibiting neuronal death mechanisms. Immunomodulatory therapies for TBI need to be developed with a goal to guide inflammation toward the reparative phenotype (99, 247). To better target such therapies, biochemical and imaging biomarkers can been considered to quantitate TBI consequences, validate preclinical research findings, and track effectiveness of therapeutic interventions in humans(201, 248–254).

## Preconditioning Penumbra Against Vulnerability To Secondary Injury

Consequences of TBI are not limited to the immediate results of primary and secondary injury mechanisms. Years after initial injury, TBI survivors can develop non-convulsive seizures/post-traumatic epilepsy (255–257) and progressive brain atrophy due to “accelerated brain aging” (258–260) that render them susceptible to further neurodegeneration (261, 262). Recurrent post-traumatic seizures, or “post-traumatic epilepsy” (PTE), are highly prevalent in TBI patients with a history of combat and are a major cause of morbidity in veterans (263). TBI severity, dural penetration, loss of consciousness, and post-traumatic amnesia are some of the risk factors that contribute to the development of PTE (263). In multiple models of TBI, it has been found that the formation of epileptogenic foci stems from excitatory/inhibitory neurotransmitter and receptor imbalances and loss of GABAergic cells (264), and tauopathy (263). Although prevention of acute seizures with anticonvulsants can manage immediate glutamate excitotoxicity, PTE tends to be refractory to current medical treatment (263). Axonal debris generated at impact (62, 209) are interrogated by microglia as early as 6 h post-TBI (265). Failure of activated microglia to phagocytose persistent axonal fragments may lead to development of TBI-induced autoantibodies (266). The persistence of axonal fragments and chronic inflammation has been documented several years after primary injury (207, 259, 267). This suggests that poor clearance of axonal debris may provoke the chronic inflammation that underlies neurodegenerative diseases (262). This may be in part due to the presence of “do not eat me” or the absence of “find me/eat me” signals, as seen in mice without CD47 (a ubiquitously expressed surface glycoprotein that provides “do not eat me” signals) which improved outcomes after TBI compared to wild type (268). Although it is clear that modulation of neuroinflammation may improve outcomes, the pharmacological and molecular tools needed to achieve this goal remain to be determined.

Beta-amyloid is another contributor to the long-term degeneration after TBI. The release of inflammasomes from activated microglia promotes seeding and polymerization of beta-amyloid (34, 210, 269) in the synapses (270). The accumulation of insoluble plaques in the extracellular space and tau neurofibrillary tangles inside neurons is already known to precede clinical symptoms of Alzheimer's disease. Tau protein deposits are also the hallmark of chronic traumatic encephalopathy (CTE), another degenerative brain disease associated with TBI. CTE tends to develop in people with a history of repetitive mild TBI such as military veterans and collision sports athletes. Retired players of the National Football League are three times more likely to die from a neurodegenerative disorder than matched controls (271). Similar to PTE, no TBI therapy has been able to address the neurodegenerative consequences modulated by the chronic inflammation that lingers years after injury.

## Adult Neurogenesis (Reparative Endogenous Neurogenesis) As A “Neuroregenerative Therapy”?

Since the discovery of CNS blast cells/neural stem cells in mammalian brain in 1989, the ability of these cells to become neurons became a topic of interest (272). Several lower-order mammals, reptiles, amphibians, and birds continue to experience neurogenesis well into adulthood. However, in adult humans, neurogenesis remains a topic of controversy. In order to understand the role of endogenous neurogenesis, it is desirable to consider how development of the cell types of the brain and the spinal cord occurs. In the mammalian fetus, NSCs are the fundamental ancestor cells for the central nervous system (CNS), as defined by their ability to self-renew and produce all three major CNS cell types: neurons, astrocytes, and oligodendrocytes (272, 273). In humans, these predecessor cells are the first neurons observed at 5-weeks after conception, even before closure of the neural tube which is lined by the neural stem cells of the ventricular zone (274). These cells are found in a layer just below the pial surface of the prospective cortex and migrate tangentially into the telencephalon (275). The two principal colonies of neural stem cells so far identified are located in the subventricular zone (SVZ) and the subgranular zone (SGZ) (276). They give rise to striatal and olfactory bulb neurons (274, 276), and hippocampal neurons, respectively.

**Figure 3:**
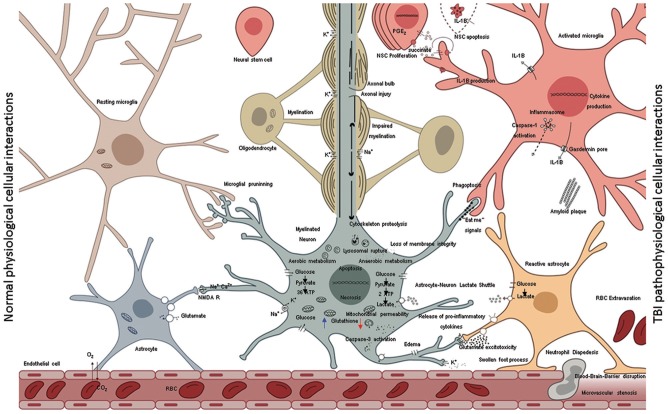
A schematic representation shows the normal cellular interactions in the intact brain (left) in contrast to neurotoxic interactions post injury (right). The intact vasculature (left bottom) is held in place by astrocytic end-feet; astrocytic blood brain barrier keeps immune cells out of the parenchyma but allows diffusion of glucose. Glucose is taken by neural cells metabolized through glycolysis in cytoplasm and oxidative phosphorylation in mitochondria. High neuronal glutathione levels (blue arrow) mitigate oxidative damage due to inherent metabolic activity. The multipartite synapses are pruned by microglia, the axons wrapped by myelin from oligodendrocytes facilitate rapid neurotransmission. Astrocytes soaking up excess glutamate in synapses, which in turns increases glucose uptake from blood. Normal microglia phagocytose various extracellular debris (including amyloid β) produce by metabolic activity. Aberrant cellular interactions after TBI (right) as consequence of mechanical forces disrupt the blood-brain-barrier causing leakage of intravascular contents into the brain parenchyma and facilitate invasion of the CNS with non-resident cells such as RBCs and neutrophils. Initial trauma causes the release of glutamate and other excitatory amino acids and potassium efflux. Lowered neuronal glutathione levels lead to deceased capacity to inhibit excitatory neurotransmission. Ions with their water shells enter astrocytes swelling the cells such that end-feet of astrocytes fail to maintain blood barrier or clear synaptic glutamate. Excess glutamate binds NMDA receptors on neurons and oligodendrocytes. Resulting neuronal depolarization and accumulation of calcium in mitochondria abolishes normal electrochemical gradient required to generate ATP. ATP dependent ion pump activity is required to work against electrochemical gradient to hyperpolarize neurons. Due to irreversible ionic imbalance membrane integrity is lost, unraveling the myelin and death of neurons and oligodendrocytes. This process is called “glutamate excitotoxicity” produces the second wave of TBI related cell death after the primary mechanical injury. Calcium pollution renders mitochondria depolarized, builds up oxidative damage, opening of the permeability transition pore, lipid peroxidation, cytochrome c release, assembly of caspase dependent proteases, and apoptosis. In addition, with injured axons calpain-induced lysosomal rupture, cathepsin-induced cytoskeletal proteolysis set into motion the self-destructive axonal degeneration. Hypoxia stabilizes HIF-1α facilitating expression of pro-inflammatory cytokine genes (IL-1β, IL-18, TNFα). Succinate acts as a signal that positively feeds inflammation. NSC disrupt such feedback and mediate inflammation resolution by rending microglia anti-inflammatory. Pro-inflammatory cytokines are released in the extracellular environment via pores (ex: IL-1β via gasdermin D) and spread inflammation to adjacent cells including mitochondrial dysfunction and secondary death of oligodendrocytes, neural stem cells and neurons. Presence of proinflammatory microglia corrupts astrocytes turning them into agents of neurotoxicity. Transient “eat-me” signals on the surface of neurons activate glial Phagoptosis leading to further loss of tissue that was otherwise intact at the time of primary injury. Proinflammatory microglia and neurons may undergo pyroptosis or other inflammatory cell death further spreading the inflammation.

Because neuronal loss is the single most important consequence of TBI, efforts to replace neurons have become a fundamental part of TBI research. In rodents, adult neurogenesis is robust even after TBI (277–279). In humans, the evidence for effective reparative adult neurogenesis is controversial, and is probably insignificant at best (280–282). NSCs persist in adult human brains and can produce astrocytes but not neurons (283). Apart from cell death, cell proliferation has also been observed after TBI. This regeneration raises the possibility of therapeutic manipulation of multipotent precursors *in situ* to repair the injured brain. The poor outcomes after TBI characterized by marked gray and white matter loss (259) do not support the notion that proliferating endogenous cells could replace lost neurons in mammals. However, physiological markers of neurogenesis and cell proliferation, measured in tissue samples one to 16 h after TBI, may indicate that the adult injured brain has the potential to replace lost cells and needs to be correlated to patients' outcomes (284). Complicating this, inflammation also contributes to the apoptosis of neural stem cells (285) and possibly oligodendrocytes (286) Figure 3. Providing neurotrophic factors, such as S100B or FGF, seems to enhance endogenous neurogenesis in experimental animals and correlates with better cognitive function (287). Failure to boost endogenous proliferation of NSCs in clinical studies (288) and the inability to produce mature neurons *in vitro* from cultured adult human NSCs reiterate that humans are incapable of adult neurogenesis (289). Proneurogenic compounds that have been found to be beneficial in preclinical TBI (290) as well as other CNS dysfunction models (291–294). Although the exact mechanism of action for these compounds is still an area of active research, the effect of proneurogenic compounds in human TBI remains to be explored. In contrast, clinical treatments with exogenous, transplanted NSCs are moving to Phase II trials, for non-TBI indications (295–300).

## Anti-Inflammatory Effects Of Transplant vs. Cell Replacement Effects

Several preclinical studies support the hypothesis that TBI-responsive neuroinflammation is a clinically relevant therapeutic target; however, few clinical trials target traumatic inflammation (117). Cell ablation pharmacological inhibition studies (114, 116, 233, 301–305) suggest that neural stem cells (NSCs), astrocytes, and activated microglia stabilize the brain lesion and prevent further neurodegeneration. However, unlike NSCs, reactive astrocytes and activated microglia are also known to exacerbate TBI (210, 270, 306, 307). It is in this context, that exogenous NSC transplantation alone may be a means to reduce neuroinflammation. Anti-inflammatory properties of mesenchyme-derived stem cells have been extensively reviewed (308) and their utility in TBI has been described elsewhere (309). Intra carotid artery delivery of human MSC was in fact found to be safe in stroke patients (310) as well as ALS (311). See Table 1 for MSC use in clinical trails.

**Table 1:** MSC trials.

	**Trial sponsor (Location)**	**Disease target**	**Cellular characteristics**	**Phase (# participants)**	**Response to treatment**	**References**
1	Neurogen Brain and Spine Institute **(India)**	Acute TBI	Autologous bone marrow mononuclear cells	Completed (43)	BMSC therapy is safe and effective on patients with severe TBI complications	NCT02028104
2	The University of Texas Health Science Center, Houston	Acute TBI	Autologous bone marrow mononuclear cells	Phase 2b-55	–	NCT02525432
	**(United States)**					
		Acute TBI	Autologous bone marrow mononuclear cells	Completed-25	Treatment is safe and effective on structural preservation and the global neuroinflammatory response	NCT01575470
3	Bioquark Inc. **(India)**	Brain death secondary to TBI	Mesenchymal stem cells	Recruiting-20	–	NCT02742857
4	Robert W. Alexander, MD, FICS	Acute TBI (concussion)	Adipose-Derived cellular stromal vascular function	Recruiting-200	–	NCT02959294
	**(United States)**					
5	SanBio, Inc. **(United States)**	Chronic motor deficit from TBI	**SB623 cells:** adult bone-marrow-derived cells that are transiently transfected with a plasmid construct encoding the intracellular domain of human Notch-1	Phase 2-52	Statistically-significant improvements in motor function, and no serious adverse events	NCT02416492
6	MD Stem Cells **(United States)**	Neurological Disorders	Intravenous and intranasal BMSC	Recruiting-300	–	NCT02795052

Similar to other chronic inflammatory diseases, addressing the impaired debris clearance by microglia may be essential in converting degeneration into regeneration (162, 312). How does chronic inflammation negatively influence TBI outcomes? As mentioned earlier, microglia are the main phagocytic cells of the brain and are responsible in part for ECF microenvironment homeostasis (313). As shown in Figure 3 following injury, the accumulation of myelin debris, beta-amyloid, and other DAMPs could impair microglia phagocytosis and exclusively activate their proinflammatory phenotype (210, 270, 314). Recent insights into biochemical differences in myelin between normal in comparison to injured subjects show how injury induced autoimmune demyelination may progress (315). This situation could potentially benefit from cell transplantation. Transplantation of human fetal NSCs within 24 h of TBI has been shown to reduce microglial pro-inflammatory activation (316) and can alleviate post-traumatic cognitive deficits (316–319). It is not known if such transplantation after PBBI would produce sustained beneficial effects. Recently, our lab demonstrated robust and durable engraftment of hNSCs when transplanted 7–10 days after the injury, in models of PBBI (309) Furthermore, the FDA has already approved of these cells for clinical trials in other CNS disorders but not yet in TBI (217, 305, 320).

One of the impediments for the long-term implementation of stem cell-based therapies is lack of insight into their mechanism of action (321). However, restorative neuroscience has been energized following the discovery of NSCs (272), their mitogens (322), their ability to be cultured from adult rodent brain (323), and embryonic (324) human brains (289). Recapitulation of human neuronal development after transplantation of human fetal neural stem cells in rodent embryos (325, 326) suggests that transplantation of NSCs could rebuild injured brains by emulating aspects of CNS development, such as tract forming and target cell innervation.

### Human Neural Stem Cells as Agents of Neuroprotection After TBI

**Figure 4:**
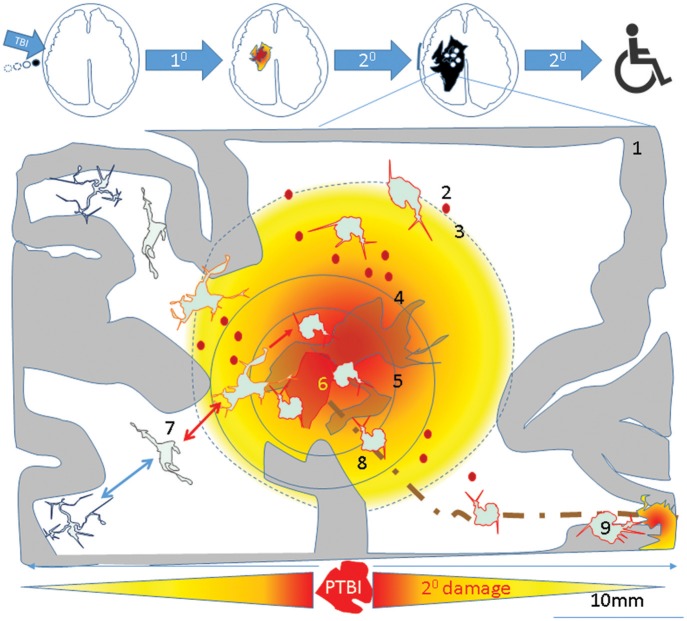
A theoretical schematic (top) shows evolution of the primary PTBI injury into disability. A PTBI brain schematic focused on inflammatory microglia over nine regions outlines possible mechanisms underlying magnification of primary injury and spread to remote sites. 1- intact brain tissue, 2-petechiae in injured brain which perpetuate blood brain barrier compromise, hemorrhage, delayed cell death, 3- a gradient of local DAMPs/NAMPs may combine with circulating inflammatory mediators (via broken BBB) to recruit and activate microglia in penumbra rendering tissue vulnerable to secondary damage, 4- region with neuronal apoptosis/pyroptosis and neural stem cell recruitment and apoptotic death., 5- regions of astrocyte destruction and reactive gliosis which stems from 6-Focal injury. The focal injury turns into a permanent cavity in part due to oncotic cell death and axonal destruction (brown dotted line). 7- Normal surveying microglial acquire various reversible/irreversible activation states as they travel along the DAMP/NAMP gradient. 8. Irreversibly activated microglia could migrate away from injury core toward remote deeper regions. 9-Pyroptosis of activated microglia in remote regions connected to injury site (via pathways that were axotomised by injury) may mediate secondary axotomy and remote neurodegeneration (210, 270).

**Figure 5:**
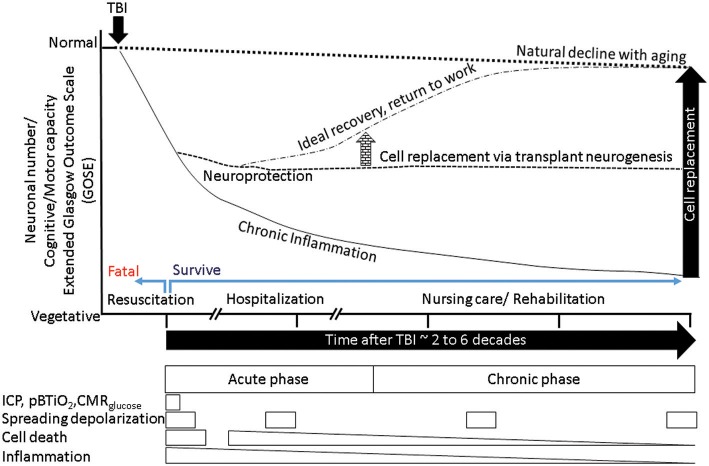
A schematic summarizes the outcome of normal aging, accelerated aging following TBI, putative mitigation of disease progression by neuroprotection, and additional benefits from cell replacement. The x-axis represents time, the y-axis represents neuronal numbers and dependent behaviors ranging from normal at the top to vegetative at the bottom. The normal aging process produces a gradual decline (dotted outline) in cognitive and motor behaviors. Following a TBI (black arrow) the process of aging is accelerated (solid downward line), with chronic inflammation and tissue loss reducing ability. Successful resuscitation can help survive otherwise fatal TBI, if post survival hospitalization produces ideal recovery then return to work is possible (dotted-dashed line), mitigation of chronic inflammation via neuroprotective agents could stem tissue loss and stabilize ability (dashed line). If the neuroprotection is mediated by neural stem cells that have the potential to replace lost cells, the new tissue in conjunction with nursing care and rehabilitation may facilitate sufficient recovery that is indistinguishable from normal aging (arrow elevating the dashed line to dashed-dotted level). The boxes below represent the transient nature of various therapeutic windows. It is evident that therapeutic windows during hospitalization are short, while those associated with disability such as post-traumatic epilepsy (PTE)/seizures depend on incidence, each event if prevented by timely intervention could mitigate further decline in ability. Acute cell death is transient, however chronic inflammation and secondary cell death that are diminishing opportunities. Hence, only acute/sub-acute neuroprotection can afford maximum benefit. However, if the cell replacement can be exploited with rehabilitation in a timely manner, there is no limit to the therapeutic window.

Successful transplantation of fetal tissue in adult rat brains (327), led to the first neuroprotective fetal cortical tissue transplants in TBI rats (328). The source of NSCs was cortical tissue (329–331). Preclinical studies of TBI showed that transplantation was acutely neuroprotective but not past 2 weeks post injury. The lack of neuronal replacement was attributed to robust host immune response. In order to overcome this limitation, researchers initiated a number of studies, including transplantation of human neuronal precursors, where experimental subjects receive immunosuppression (205). One of the mechanisms of action for hNSC transplants was elucidated in a multiple sclerosis model. In that study, NSCs appeared to sense the extracellular succinate that accumulates in the chronically inflamed CNS and ameliorated neuroinflammation via succinate-SUCNR1-dependent mechanisms (332). Consistent with these findings iPSC derived NSC as well as oligo precursor transplants are reported to spare tissue in rodent spinal cord injury model (333). Such tissue sparing occurs following inflammation resolution. As outlined in Figure 4, in the absence of timely resolution of injury induced activated microglia, the injury is magnified over time and space producing progressive increased tissue destruction in part *via* microglial pyroptosis (210) and facilitates worse outcomes such as antibody generation against cellular breakdown products (266) and neurodegeneration (Figure 5). Neural stem cell transplants could confer neuroprotection to alleviate such tissue loss and lead to a desired outcome via inhibiting microglial pyroptosis, disrupting the succinate based inflammation amplification, and promoting phagocytosis by surviving activated microglia (Figure 5). Perhaps all stem cells confer neuroprotection via efferocytosis (334).

### Cell Replacement

Transplanted NSC-derived neurons can integrate and contribute electrophysiologically in both sham as well as injured rodent and primate brains (321, 335–339). However, previous studies have reported limited neuronal replacement after hNSCs transplantation in rat models of TBI (318). Recently our lab and others have shown robust and durable engraftment of hNSCs with delayed differentiation into mature neurons, for as many as 20% of transplanted cells, up to 16 weeks in a rodent PBBI model. Nevertheless, their integration into injured rat CNS and contribution to reversal of TBI induced motor and cognitive deficits has yet to be fully demonstrated (309, 340). Assessing electrophysiological properties and their contribution to amelioration of TBI-induced deficits would provide crucial mechanistic insight. It is not yet known if it is possible and/or necessary to guide transplant-derived neurons to a specific target (such as anterior horn cells, or substantia nigra) and how this can be done. The use of clinically relevant neurogenic compounds could be key to assist in targeting the migration of transplant-derived neurons. In primates with SCI, researchers have found that transplanted NSI 566 cells can be harnessed to restore lost function 9 months after grafting by differentiating into neurons and supportive glia (339). If cell replacement is indeed achieved it could positively steer outcomes (Figure 5).

The current literature suggests that NSC mediated neuroprotection (332, 333) could be achieved more easily than cell replacement, especially in severe TBI. It is true that the incidence of severe TBI is small and cell therapy to treat will not be appealing to mild and moderate TBI patients. However, the costs associated with TBI requiring hospitalization necessitate use of cell therapy to treat sever TBI (18, 341). Following the discovery of the mechanistic insights, “the neuroprotective factor” could be delivered via non-cell transplantation means even for less severe TBI where cell therapy is not warranted.

## Rationale For Use Of hNSC To Treat TBI And Guidelines For Cell Therapy

After examining several RCTs and gaining insights into their failure to confer neuroprotection, (11, 90, 114), identification of anti-inflammatory mechanisms as leading neuroprotective agents, the neuroprotective properties sub-acute use of human NSCs (332, 333, 342)is worthy of exploration in TBI. NSCs could potentially mitigate secondary damage by (1) reducing inflammation; (2) promoting regeneration with appropriate pharmacological interventions (e.g., drugs promoting neurogenesis such as NSI-189) and rehabilitative measures; and (3) Slowing down TBI-induced delayed disability. Accomplishing this set of objectives in itself would be an important goal of NSC therapy. As of mid-2018, a total of 316 patients with various reported CNS disorders have received clinical grade hNSC transplants (Table 2). None of these patients had any safety issues yet. Neuralstem Inc., has sponsored phase I and phase II clinical trials evaluating hNSC transplantation as a potential therapy for ALS (354). In these, a *post hoc* analysis compared ambulatory limb-onset ALS participants who were administered open-label intraspinal hNSC and followed for up to 3 years after transplant. Due to lack of controls, participants in these phase 1 and 2 trials were matched to subjects from the Pooled Resource Open-Access ALS Clinical Trials (PRO-ACT) and ceftriaxone datasets to provide required analyses in order to inform future clinical trial designs. The ALS Functional Rating Scale revised (ALSFRSR) and a composite statistic combining survival and functional status (ALS/SURV) were assessed to monitor changes in function. Results from These Ph1/2 studies revealed significantly improved survival and function (346) when compared to historical datasets. In another study where non-ambulatory ALS patients received either unilateral or bilateral injections, no increase of disease progression after the transplants was observed for up to 18 months after surgery. Rather, two patients showed a transitory improvement of the subscore ambulation on the ALS-FRS-R scale (from 1 to 2). A third patient showed improvement of the MRC score for tibialis anterior, which persisted for as long as 7 months. Three of the patients died due to disease progression (353). More recently a study of stereotactic, intracerebral injection of CTX0E03 neural stem cells from ReNeuron into patients with moderate to moderately severe disability as a result of an ischemic stroke has progressed from a Phase I to Phase IIb as the clinical endpoints are being met albeit slower than expected (295, 299, 300).

**Table 2:** Human fetal neural stem cells in Clinical Trials.

	**Trial sponsor (Location)**	**Disease target**	**Cellular characteristics**	**Phase** **(#. treated)**	**Response to treatment**	**References**
1	City of Hope (CA, USA)	recurrent high grade gliomas	carboxylesterase-expressing neural stem cells	phase I (15)	Initial safety and proof of concept regarding the ability of NSCs to target brain tumors	NCT01172964 (343–345)
2	Neuralstem Inc. (MD, USA) in collaboration with Emory University Atlanta, Georgia, United States, Massachusetts General Hospital Boston, Massachusetts, United States, University of Michigan, Ann Arbor, Michigan, United States	ALS	Eight wk fetal-derived neural stem cells	phase I (18)	Safe	NCT01348451 (346)
				phase II (18)	Improved survival compared to standard treatment	NCT01730716
		chronic spinal cord injury		phase I (8)	On going	NCT01772810 and NSI website (347)
		stroke		phase I/II (9)	Safe with behavior modification	NSI website
3	ReNeuron Ltd. (UK) Division of Clinical Neurosciences, Glasgow Southern General Hospital, Glasgow, UK, G51 4TF,ReNeuron, Queen Elizabeth Hospital, Birmingham, UK, NHS Southern General Hospital, Glasgow, UK, G51 4TF, Kings College Hospital London, UKUniversity College London Hospital London, UK, Royal Victoria Infirmary Newcastle, UK Nottingham City Hospital Nottingham, UK Salford Royal NHS Foundation Trust Salford, UK Royal Hallamshire Hospital Sheffield, UK Southampton Hospital Southampton, UK	stroke	12 wk fetal cortex derived, genetically modified CTX0E03 neural stem cells	phase I (12)	Safe	NCT01151124 (299)
				phase II (41)	Safe with behavior modification slightly delayed than expected.	NCT02117635
4	ReNeuron, Division of Clinical Neurosciences, Glasgow Southern General Hospital, Glasgow, UK, G51 4TF	lower limb ischemia		phase I (9)		NCT03333980
5	Stem Cells Inc. (CA, USA)	neuronal ceroid lipofuscinosis	16 wk fetal-derived neural stem cells	phase I (6)		NCT00337636 (348)
		cervical spinal cord injury		phase II (50)		
		macular degeneration		phase I/II (15)		
		thoracic spinal cord injury		phase I/II (12)		NCT01321333 (349, 350)
		Pelizaeus-Merzbacher disease		phase I (4)		NCT01005004
6	TRANSEURO (UK) STEM-PD	Parkinson's disease	fetal-derived dopaminergic cells	phase I (40)		NCT01898390 (351, 352)
7	Azienda Ospedaliera Santa Maria, Eastern Piedmont University, Novara and Terni Hospital, Terni,Italy.	ALS	8 wk fetal-derived neural stem cells	phase I (3)		EudraCT:2009-014484-39 (353)

## Pathway To Address Unmet Patient Need, Clinical Trials, To Arrive At Proven Treatments

The previous sections suggest that the unit of intervention for TBI should be at the cellular level i.e., at the unit of life. However, it is important to be wary of moving too hastily. The compelling unmet TBI medical need and desperation on the part of patients, in the absence of multicenter clinical trials, can lead to unproven therapies being administered to patients. Three such cases of unproven stem cell therapies (mix of multiple fetal NSCs or MSCs and NSCs) have been documented (355–357). Fortunately, all of the issues could be resolved by corrective measures i.e., removal of the transplanted cells. The aforementioned events have their roots in premature and unapproved use of treatments that were initiated by investigator/patient. “Stem cell tourism” that exploits the therapeutic hope of patients and families with incurable neurological diseases can jeopardize the legitimacy of stem cell research. Julian et al posit that an improvement in education, regulation, legislation, and involvement of authorities in global health in neurology and neurosurgery is required to prevent such exploitation (358) (Table 3).

**Table 3:** Unproven application of cell therapy.

1	Moscow Hospital	Ataxia telangiectasia (AT)	8–12 wk aborted fetal periventricular tissue isolated from fresh-autopsy cultured for ~16 days, 1–2 fetus/procedure, 3 procedures total	1	Tumor formation	(355)
2	Commercial stem-cell clinics in China, Argentina, and Mexico.	Residual deficits from an ischemic stroke	the infusions were described as consisting of mesenchymal, embryonic, and fetal neural stem cells	1	Debilitating glioproliferation	(356)
3	“stem cells” at a clinic in Georgia, USAPrivate clinic,	Exudative macular degeneration	autologous adipose tissue-derived “stem cells”	1	Bilateral Retinal Detachments	(357)
4	Thailand-Canada	Lupus nephritis	autologous CD34+ hematopoietic stem cell transplantation, mobilized with GCF and collected from peripheral blood	1	angiomyeloproliferative lesions	(359)
5	Multiple US based Stem cells clinics in FL, CT and MD	Patient for each of the following conditions: Two for non neovascular AMD, and one for Quiescent neovascular AMD	autologous adipose tissue-derived “stem cells”	3	Loss of vision	NCT01736059: NCT02320812, (360), NCT01920867, NCT0024269
6	Stem Cell Ophthalmology Treatment Study (SCOTS) FL,CT,MD	Stargardt's macular dystrophy	autologous bone marrow-derived stem cells in the right eye	1	recurrent retinal detachment with proliferative vitreoretinopathy.	(361)
7	Stem Cell Ophthalmology Treatment Study (SCOTS) FL,CT,MD	optic neuropathy	autologous bone marrow-derived stem cells	1	Improved vision	NCT 01920867 (362)
Unproven stem cells use with beneficial vs. debilitating consequences				1 success vs. 8 failures		

## Conclusion

For over 30 years of TBI research, neuroprotection via RCTs has been elusive. Progressive tissue loss in severe TBI is an unmet need that turns TBI into a disease process with no hope for recovery. Analysis of the trial failures has led to insights into the mechanisms that need to be targeted, specifically neuroinflammation. Preclinical animal model studies that recapitulate human severe TBI have led to the identification of mechanisms underlying the vulnerability of the penumbra and evaluating the extent of penumbra sparing will likely give insights into the neuroprotective ability of an intervention. Additionally, the continued exploration of neural stem cells transplantation, which was bolstered by initial efforts with fetal cortical tissue transplants that were neuroprotective, resulted in the discovery that cell transplants can resolve inflammation via disruption of proinflammatory pyroptotic signaling and without interfering with activated glial functions such as phagocytosis. Multiple independent studies in a variety of CNS conditions suggest use of clinical trial grade human neural stem cells which have been found to be safe and meeting the clinical end points. Thus, the rational for using human neural stem cell based transplantation for TBI is well supported as both enduring neuroprotection and cell replacement can be achieved with single agent.

## Author Contributions

AK, AC, DC, SL, MS, KR, HP, LD, and JW reviewed the preclinical articles and prepared the draft, AM wrote the section on trial cost. AA, SY, ZH, SL, AR, RB, and RT worked clinical trials articles and prepared draft. SN, HP, and MS generated the data for figures. LL, AA, SY, SG, and RB conceived and revised the draft to produce final manuscript. AK produced original artwork to encapsulate the literature on cellular interactions with RB and SG.

### Conflict of Interest Statement

The authors declare that the research was conducted in the absence of any commercial or financial relationships that could be construed as a potential conflict of interest.
